# The Bioavailability and Biological Activities of Phytosterols as Modulators of Cholesterol Metabolism

**DOI:** 10.3390/molecules27020523

**Published:** 2022-01-14

**Authors:** Xiang Li, Yan Xin, Yuqian Mo, Pavel Marozik, Taiping He, Honghui Guo

**Affiliations:** 1 Department of Nutrition, School of Public Health, Guangdong Medical University, Zhanjiang 524023, China; gdydzds@gumu.edu.cn; 2 Dongguan Key Laboratory of Environmental Medicine, School of Public Health, Guangdong Medical University, Dongguan 523808, China; xy1999@gdmu.edu.cn (Y.X.); moyuqian@gdmu.edu.cn (Y.M.); 3 Laboratory of Human Genetics, Institute of Genetics and Cytology of the National Academy of Sciences of Belarus, 220072 Minsk, Belarus; P.Marozik@igc.by

**Keywords:** phytosterol, cholesterol, low density lipoprotein, bioavailability

## Abstract

Phytosterols are natural sterols widely found in plants that have a variety of physiological functions, and their role in reducing cholesterol absorption has garnered much attention. Although the bioavailability of phytosterols is only 0.5–2%, they can still promote cholesterol balance in the body. A mechanism of phytosterols for lowering cholesterol has now been proposed. They not only reduce the uptake of cholesterol in the intestinal lumen and affect its transport, but also regulate the metabolism of cholesterol in the liver. In addition, phytosterols can significantly reduce the plasma concentration of total cholesterol, triglycerides, and low-density lipoprotein cholesterol (LDL-C), with a dose-response relationship. Ingestion of 3 g of phytosterols per day can reach the platform period, and this dose can reduce LDL-C by about 10.7%. On the other hand, phytosterols can also activate the liver X receptor α-CPY7A1 mediated bile acids excretion pathway and accelerate the transformation and metabolism of cholesterol. This article reviews the research progress of phytosterols as a molecular regulator of cholesterol and the mechanism of action for this pharmacological effect.

## 1. Introduction

Phytosterols, encompassing plant sterols and stanols, are natural steroids that are widely found in different parts of plants (including roots, stems, leaves, flowers, fruits, and whole grasses) and are an important part of plant cell membranes. People ingest 100–400 mg/d phytosterols, mainly from vegetable oils, bread, cereals, nuts, and vegetables [1,2,3,4]. Phytosterols have been reported to lower cholesterol and low-density lipoprotein cholesterol (LDL-C) plasma levels and may have clinical application for the prevention of non-alcoholic fatty liver disease (NAFLD) and cardiovascular diseases (CVDs).

Currently, the physiological functions of phytosterols can be reflected in many aspects, such as an antioxidant function [5], anti-inflammatory and antipyretic effects [6], and a hormone-like effect [7]. Their most remarkable function is to reduce the absorption of cholesterol as well as the concentration of LDL-C [8,9]. Phytosterols have a similar structure to cholesterol and, in the intestine, will compete and hinder the absorption of cholesterol, thereby reducing the level of LDL-C in the plasma. The work of Agren et al. has shown that rheumatoid arthritis patients consuming 732 mg of phytosterols in their daily diet saw an obvious reduction in total cholesterol and LDL-C [10]. In addition to the above physiological functions, phytosterols also play many other roles in promoting human health. For example, they can improve insulin resistance [11] and lipid metabolism [12], and can reduce cancer risk [13], Alzheimer’s disease [14], as well as the risk of atherosclerosis related CVDs [15]. Fundamentally, the above-mentioned preventive effects can also be attributed to the ability of phytosterols to lower cholesterol. Because of their many beneficial functions to the human body, phytosterols are widely used in functional foods.

Abnormal cholesterol metabolism is an important factor in many chronic diseases. Although the cholesterol-lowering effect of phytosterols has gradually attracted attention, their mechanism of action is still unclear. To better understand the molecular mechanisms by which phytosterols affect cholesterol absorption and metabolism, this review focuses on recent clinical trials involving phytosterol interventions and, using NAFLD and CVDs as examples, explores the protective role of phytosterols, including the modulation of exogenous cholesterol absorption and endogenous cholesterol synthesis. This study provides a theoretical reference to further encourage the population to consume phytosterol-rich foods with the goal of reducing the risk of hypercholesterolemia-related diseases.

## 2. Phytosterols Chemistry and Dietary Sources

**Figure 1:**
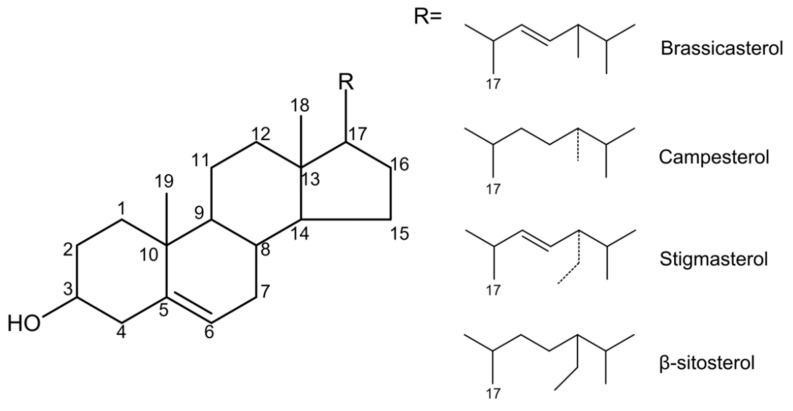
The main chemical structure of different kinds of phytosterols.

Phytosterols are naturally occurring compounds in the triterpene family that are widely found in plants. The structure of phytosterols is very like cholesterol, so the two have similar physicochemical properties. The difference is that the C-24 of phytosterols contains methyl or ethyl groups [16]. Based on the difference in substitution on the side chains of C-4 and C-24, the difference in the degree of unsaturation of the side chain and ring, and the diversity of the combination of alcoholic hydroxyl groups at the C-3 position with other compounds, more than 250 phytosterols have been reported [7]. The main chemical structures among them are four ring structures and C-17 side chains (Figure 1).

Most plants in nature contain phytosterols, but the content of phytosterols differs largely among the different parts of the plant. The oils, nuts, beans, and seeds of plants are good sources of phytosterols. Although phytosterols can be detected in fruits and vegetables, their content is relatively low. In olive oil, the content of total phytosterols is 235.9 ± 11 mg/100 g, whereas in the potato, the content of total phytosterols is only 4.3 ± 0.2 mg/100 g [2]. In higher plants, the main phytosterols are β-sitosterol, campesterol, and stigmasterol, and the content of these three sterols can reach more than 80%. For example, in *Arabidopsis thaliana*, the main sterol is β-sitosterol, accounting for 64%, while other sterols are found, including campesterol (11%), stigmasterol (6%), isofucosterol (3%), and brassicasterol (2%) [17]. In olive flowers, the total sterol level rises from the early stage to the flowering stage and during this period β-sitosterol and cholesterol accumulate, with the content of β-sitosterol even reaching 96% [18]. In peanuts, the seed heart, kernels, and seed coats contain β-sitosterol, campesterol, and stigmasterol with contents of 82.29%, 86.39%, and 94.25%, respectively [19].

Currently, several countries recommend an intake of 1.5–3 g/d of phytosterols to reduce the risk of coronary heart disease [20]. The results of population-based surveys showed that the intake of phytosterols in the city of São Paulo was about 100 mg/d, which was lower than 170 mg/d in the United States. The average daily intake in Poland is 256 mg/d [4] and in Belgium it is 280 mg/d [21]. The dietary phytosterol intake of Chinese residents is about 392.3 mg/d [22]. In Mexico, the intake of phytosterols can reach 400 mg/d [23]. However, the current intake is far less than the recommended amount to prevent hypercholesterolemia and cardiovascular diseases. Considering the health-promoting effects of phytosterols, people can increase phytosterols intake by consuming phytosterol-rich foods or dietary supplements, especially for people with hypercholesteremia and high dietary cholesterol intakes.

## 3. Bioavailability of Phytosterols

In order to evaluate the health-promoting effects of phytosterols more scientifically, it is very important to fully understand the pharmacokinetics of phytosterols, because only bioactive substances with certain bioavailability can exert their biological activities in the human body.

**Figure 2:**
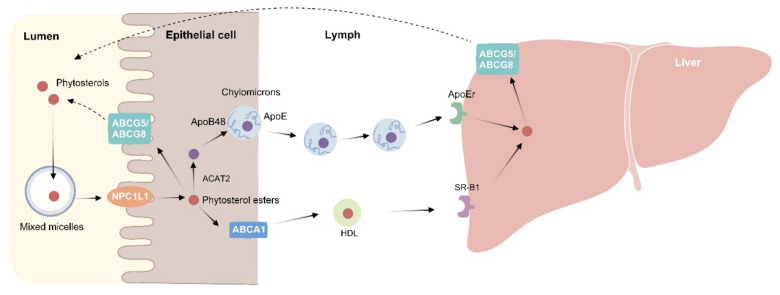
Phytosterol metabolism. In the intestinal lumen, dietary fat, cholesterol, and bile acids mix to form micelles. Phytosterols compete with cholesterol to enter the micelles. Free phytosterols are absorbed by intestinal epithelial cells via NPC1L1 and then esterified by acyl-CoA: cholesterol acyltransferase isoform 2 (ACAT2) to cholesteryl esters and incorporated into chylomicron. The unesterified phytosterols are secreted back into the intestinal lumen via ATP binding cassette (ABC) G5/8. After chylomicron enters the circulation, it transfers free fatty acids to the peripheral tissues, and its residues are taken up by the liver via *ApoE*-dependent receptor (ApoEr). Phytosterols in the liver can also be transported back into the intestinal lumen via the *ABCG5/8* transporter at the hepatobiliary interface. On the other hand, phytosterols from intestinal epithelial cells can also enter HDL through ABCA1 at the basolateral membrane. HDL is recognized by the scavenger receptor class B type 1 (SR-B1) receptors in the liver and then absorbed into the liver.

The bioavailability of phytosterols depends on many factors, including some intestinal transporters, their different molecular types, and genetic factors. Phytosterols and cholesterol have similar chemical structures, but the intestinal absorption rate of phytosterol is much less than that of cholesterol. Only less than 5% of phytosterols can be absorbed [24], while cholesterol can be absorbed at 50–60% [25]. Phytosterols follow food into the digestive tract, bind to the sterol transporter Niemann-pick C1-like 1 (NPC1L1) located in the apical membrane of intestinal cells in the intestinal lumen (Figure 2), and are then absorbed by intestinal epithelial cells [26]. However, free phytosterols are excreted into the intestinal lumen by ATP binding cassette (ABC) G5/8 in the intestinal cells. There are a small number of phytosterol molecules that can circumvent this mechanism and are then carried into the circulation by lipoproteins. The quantity distribution of the lipoproteins is similar to that of cholesterol, so that most of the phytosterol molecules circulate in LDL particles (70–80%). Another process of phytosterol metabolism is to combine with the ABCA1 transporter in the basolateral membrane of intestinal cells and mobilize to become part of HDL particles. After being transported to the liver, phytosterols can also be transported back to the intestine by *ABCG5/8* protein at the hepatobiliary interface [27].

In addition to the *ABCG5/8* transporter, the bioavailability of phytosterols is also directly or indirectly influenced by several other proteins and genes, which can affect the transport of phytosterols across intestinal cells. For example, phytosterols will be absorbed by NPC1L1 in the intestinal lumen. Therefore, genetic mutations in NPC1L1 may also influence the bioavailability of phytosterols. Furthermore, Tammi et al. and Lupattelli et al. have observed that apolipoprotein E (*ApoE*) 3/4 or 4/4 allele participants have significantly higher efficiency of phytosterol absorption than *ApoE*3/3 allele participants [28,29]. However, no clear mechanism has yet been proposed to explain how *ApoE* affects phytosterol absorption. Chan et al. found that *ApoE* phenotype affected circulating phytosterol concentrations [30], but another research found that *ApoE* phenotype did not alter plasma campesterol concentration [31]. Thus, whether *ApoE* affects the bioavailability of PS needs further research. Moreover, the molecular structure can also affect the bioavailability of phytosterols. There are two types of free phytosterols: sterols and stanols. The former mainly includes sitosterol, campesterol, and stigmasterol; the latter mainly includes sitostanol and campestanol. Besides, there are combined forms of sterol esters and sterol glycosides. Borel et al. demonstrated that the absorption efficiency of plant sterols and plant stanols in the body is about 0.04–1.9% (sitosterol 0.51%, campesterol 1.9%, sitostanol 0.04%, and campestanol 0.16%) [32]. The absorption efficiency of plant sterols can reach 10 times that of plant stanols. Based on this evidence, it is obvious that the type of molecule has an impact on the bioavailability of phytosterols.

Single nucleotide polymorphism (SNP) was found to significantly affect the blood phytosterol concentration [33], for example, the SNPs of NPC1L1 can significantly increase the serum PS level [34], demonstrating that genetic factors can affect the bioavailability of PS.

In view of the low bioavailability of phytosterols, it is hoped that chemical or physical modification can increase their bioavailability. Chemical modification has focused mainly on esterification, while physical modification has been achieved by microencapsulation [24]. Recently, Jones et al. developed a nano-scale delivery system to improve the bioavailability of lipophilic biologically active substances [35]. By customizing a delivery system, the biologically active substance can be delivered to its specific absorption site. The advantage of this system is that it can reduce the loss of biologically active substances before reaching the absorption site in order to improve the bioavailability of the compound. However, the system still has some shortcomings, and toxicological evaluation of the complex food matrix and system is still lacking. The few existing results show that phytosterols can be obtained from various food matrices to reduce the absorption of cholesterol, and the increase in the bioavailability of carotenoids can be achieved through food processing [8,36,37]. Further research directions point to examining the influence of the food matrix on the nano-delivery system, the toxicological evaluation of the nano-delivery system on both the food matrix and environment, and the influence of food processing on the bioavailability of phytosterols. When investigated together, this evidence highlights the great significance of improving the bioavailability of phytosterols.

In recent years, it has been shown that PS may play a pro-atherosclerotic role in humans. Bao et al. hypothesized that sitosterol induces accelerated death of macrophages, which induces rupture of atherosclerotic plaques and ultimately leads to development of cardiovascular disease [38]. Similar dangerous effects have been found in vivo and in clinical cases [39]. In contrast, Genser et al. showed that no significant relationship was found between circulating PS and its ratio to cholesterol and the risk of cardiovascular disease [40]. Recent clinical studies of dietary interventions have also shown that PS intake may have beneficial effects on cardiovascular health [41,42]. Silbernagel et al. found that mutations in these loci, *ABCG8* and ABO led to increased circulating PS concentrations and regulated total cholesterol and LCL-C levels [43], Therefore, it is controversial whether PS has pro-atherosclerosis effects, but in general, a moderate increase in PS bioavailability is clearly beneficial to human health.

## 4. Cholesterol-Lowering Effect of Phytosterols in Patients with Hypercholesterolemia-Related Diseases

Nowadays, it is becoming increasingly appreciated that phytosterols do enter the circulation. Moreover, although circulating concentrations are very low compared to cholesterol, they are taken up by different tissues [44] and may affect the pathological processes of some diseases, such as NALFD and CVDs.

NALFD is the most common chronic liver disease worldwide, with a broad spectrum ranging from simple steatosis to the advanced stages of liver fibrosis and cirrhosis [45]. Until now, the progression of NAFLD is not exactly understood, and it is believed to have multiple pathogenic mechanisms. Risk factors such as diabetes, obesity, and insulin resistance may all lead to the progression of NAFLD. These metabolic diseases are associated with the increase of a variety of cytokines that mediate inflammation, and the increase in these inflammatory factors will promote the progression of NAFLD and liver damage [46].

A multi-hit theory has gradually emerged in recent years and proposes that lipotoxicity, mitochondrial dysfunction, endoplasmic reticular stress, inflammation, overnutrition, and intestinal flora disorders are important risk factors for the development of NAFLD [47]. Among them, lipotoxicity refers to the abnormal accumulation of lipids in non-fat tissues due to various reasons, which in turn has toxic effects on cells [48]. Currently, it is recognized that lipid toxic molecules, mainly cholesterol and free fatty acids (FFAs) and their derivatives, have a great negative impact on the occurrence and development of NAFLD [49].

Lipid rafts, which exist in the membrane lipid bilayer and can transmit some signals, are formed by cholesterol, sphingomyelin, and glycosphingolipids [50]. The ratio of cholesterol to phospholipid on the cell membrane is maintained at an appropriate level, but when the ratio is unbalanced, the structure of the lipid raft breaks down and the fluidity of the cell membrane is also reduced.

Increased cholesterol on the mitochondrial membrane will reduce membrane fluidity, resulting in a decrease in carrier protein activity, limited α-ketoglutarate transport, and reduced glutathione (GSH) transport from the cytoplasm to the mitochondria. This then leads to the consumption of mitochondrial GSH, which is the main antioxidant in the mitochondria. The consumption of GSH results in mitochondria that are more sensitive to oxidants, and can even promote mitochondria to produce reactive oxygen species (ROS) and lipid peroxidation [51]. Free cholesterol (FC) overload can damage the activity of the endoplasmic reticulum membrane Ca^2+^-ATPase (SERCA), induce endoplasmic reticular stress (ERs) and unfolded protein response (UPR), activate the expression of pro-apoptotic factor caspase-3, and then induce hepatocyte apoptosis [52]. It plays an important role in the progression of NAFLD. In addition, the accumulation of FC also promotes the production of toxic oxysterols and induces adipose tissue dysfunction to directly or indirectly damage hepatocytes [53].

The enzyme 3-hydroxy-3-methylglutaryl coenzyme A reductase (HMGCR) is a key rate limiting enzyme in the de novo cholesterol synthesis pathway. Dysregulation of hepatocyte cholesterol homeostasis occurs in NAFLD patients. In the case of intracellular cholesterol overload, SREBP-2, one of the main nuclear factors regulating cholesterol metabolism, is abnormally activated under the action of proinflammatory factors, resulting in the increased expression and activity of LDL receptor (LDLR) and HMGCR, which in turn results in excessive accumulation of cholesterol in the liver, accelerating the development of fatty liver disease [54]. Cholesterol 7 α-hydroxylase (CYP7A1) and sterol 27-hydroxylase (CYP27A) convert cholesterol to bile acids (BAs) through classical and alternative pathways, respectively [55]. Fibroblast growth factor 19 (FGF-19) inhibits cholesterol synthesis of BAs through P450 7A1 when it circulates into hepatocytes [56]. Decreased levels of CYP7A1, CYP27A, and FGF-19 have been observed in NAFLD patients, resulting in decreased cholesterol degradation, further increasing FC accumulation in the liver and accelerating the progression of NAFLD [57].

Despite the low bioavailability of phytosterols, Feng et al. have shown that phytosterols can reduce hepatic triglyceride and cholesterol accumulation in mice fed a high-fat Western-style diet [58], indicating that phytosterols may exert their biological activity in the liver.

Laos et al. found that phytosterols can not only reduce liver steatosis, but may also reduce liver triacylglyceremia caused by excessive cholesterol load [59]. In particular, long-term consumption of foods rich in phytosterols can effectively reduce various risk factors for NAFLD, such as serum triglycerides (TG) and free fatty acids concentration, as well as increase bile acids synthesis and other mechanisms to reduce the risk and prevent the development of NAFLD. In a double-blind clinical trial in which 38 patients with NAFLD were given 1.6 g of phytosterol supplementation daily for 8 weeks, it was found that phytosterol intervention could effectively reduce the concentration of risk factors such as LDL-C, TNF-α, ALT, and AST [60]. In addition, the authors speculated that the cholesterol-lowering function of phytosterols may be achieved through reducing the esterification rate of cholesterol in intestinal epithelial cells. Moreover, Gumede et al. fed rats with a high-fructose diet to induce NAFLD, and then intervened with β-sitosterol and found that the fatty liver degeneration caused by the high-fructose diet and the evolution of NAFLD to NASH were both prevented by β-sitosterol [61]. Recently, Song et al. have investigated the effects of plant sterol esters (PSE) on NAFLD in rats fed a high-fat diet [62]. After 12 weeks of gavage, it was found that the relative abundance of *Bacteroides* and *Anaerobic* bacteria was significantly increased compared with the rats fed a normal chow diet, suggesting that PSE may regulate the intestinal flora to protect against NAFLD. In addition, Song et al. have found that the combined effect of phytosterols and n-3 fatty acids (DHA and EPA) in the treatment of NAFLD exhibited a more significant therapeutic effect than a single dose of phytosterols, which may be due to the synergistic effect of phytosterols and DHA+EPA [63]. Furthermore, it is suggested that phytosterols can work together with other biologically active substances to enhance the therapeutic effect on NAFLD. Because of differences in ethnicity, animal experiments cannot fully represent the results of population experiments. There is currently a lack of long-term, large-scale clinical trials to prove that phytosterols can reduce NAFLD in humans.

As we all know, if the cholesterol balance in the body is broken, the concentration of cholesterol in the plasma will increase, and excessive cholesterol will accumulate in different tissues, leading to an increased risk of atherosclerosis and CVDs. Currently, it is recognized that one of the most important functions of phytosterols is to lower serum LDL-C, which is achieved at least in part by reducing the absorption of intestinal cholesterol.

CVDs are the leading cause of death globally, and include coronary heart disease (CHD), cerebrovascular disease (stroke), and peripheral vascular disease [64]. One of the underlying risk factors for CVDs is dyslipidemia, which can lead to increased concentrations of circulating blood cholesterol and triglycerides and is characterized by elevated LDL-C. It is often combined with low concentrations of high-density lipoprotein cholesterol (HDL-C) and elevated TG. Numerous trials have suggested that, based on its effect on LDL-C, consumption of phytosterol supplements could lower the risk of CVDs [65] (Table 1). A randomized controlled trial in which 92 asymptomatic subjects ingested 3 g of plant stanols daily through a rapeseed oil-based enriched spread over 6 months, displayed a 10% decrease in LDL-C and a reduction of arterial stiffness in small arteries—a marker of subclinical atherosclerosis [66]. As consumption of phytosterols modestly elevates endogenous cholesterol synthesis, their cardio-protective effects may be further promoted by elevation of cholesterol synthesis markers. Notably, the hypocholesterolemic effect of phytosterols on LDL-C was most pronounced in individuals with low basal and endogenous cholesterol synthesis [67].

However, clinical data are still lacking to demonstrate the potential effects of phytosterol supplementation on hard clinical endpoints of CVDs. Despite plasma cholesterol reduction by phytosterol intake, since CVDs are multi-factorial, no definite conclusion can be drawn. This can be addressed through results from human trials with clinical endpoints or lesion size development from animal studies with different genetically modified mouse models.

## 5. The Underlying Mechanism of Phytosterols in Regulating Cholesterol Homeostasis

### 5.1. Phytosterols Regulate the Absorption of Cholesterol in the Gut

It is well known that the gut is the main site of cholesterol absorption. Some of the cholesterol in food and bile is reabsorbed in the intestinal lumen, while the rest is excreted in the feces.

**Figure 3:**
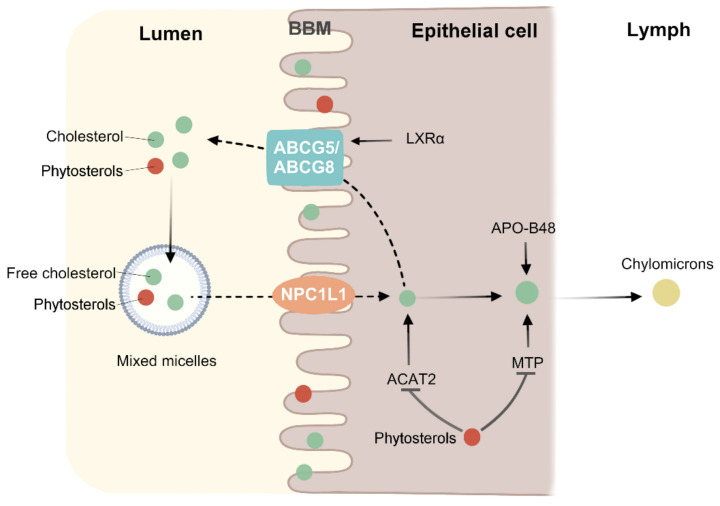
Phytosterols inhibit cholesterol absorption. Cholesterol and phytosterols have similar chemical structures, and thus have similar metabolic mechanisms. Apolipoprotein B48 (APO-B48) and microsomal triglyceride transfer protein (MTP) can incorporate cholesterol esters into chylomicron. *ABCG5* and *ABCG8* genes may be upregulated by Liver X receptor α (LXRα) in a high cholesterol environment. Phytosterols compete with cholesterol in micelles in the intestinal lumen and brush border membrane (BBM), thereby reducing cholesterol absorption. By affecting the expression of ACAT2 and MTP, the cholesterol esterification and the amount of cholesterol entering chylomicron were reduced.

The absorption of cholesterol in the human gut is a complex process involving many interrelated physiological pathways. First, unesterified cholesterol enters the bile acid micelles, by which it is transported to the brush edges of intestinal cells. It is then absorbed into intestinal epithelial cells by NPC1L1. Cholesterol entering the intestinal epithelium is esterified by associated proteins such as acyl-CoA: cholesterol acyltransferase isoform 2 (ACAT2) and then secreted by microsomal triglyceride transfer protein (MTP) into the new chylomicrons in the lymph [74]. In contrast, *ABCG5/8* on the brush edge of intestinal cells promotes cholesterol exudation from the intestinal cells into the lumen by the formation of heterodimers [75] (Figure 3). Thus, the absorption of cholesterol in the intestinal tract is not a simple protein transport, but a complex multi-step process regulated by multiple genes.

Phytosterols and PSE are present mainly as fatty acid esters, hydroxycinnamic acid esters, and glycosides [76]. Within the gastro-intestinal tract, all ester bonds are cleaved by specific enzymes, resulting in the formation of free phytosterols and stanols. The free phytosterols are subsequently incorporated into mixed micelles, and because phytosterols are more strongly hydrophobic than cholesterol, the micelles have a higher affinity for phytosterols, thus phytosterols will replace the cholesterol in the micelles, reducing the absorption of cholesterol in the intestines and increasing the excretion of cholesterol. Like cholesterol, phytosterols are taken up from the mixed micelles into enterocytes via the NPC1L1 receptor, located at the apical membrane [77]. NPC1L1 is the pharmacological target for ezetimibe, which efficiently lowers intestinal absorption of both cholesterol and phytosterols [78]. At the same time, this mechanism implies that phytosterols should be consumed with foods containing cholesterol in order to reduce cholesterol absorption [79]. However, there is evidence that the frequency of daily phytosterol consumption does not change the cholesterol-lowering effect, and the effect of a single intake of the same dose is the same as that of multiple meals [80,81], while the results of Doornbos et al. showed that a single-dose of PS with a meal had more significant LDL-C lowering effect than that without a meal [82]. In addition, PS is excreted more rapidly than cholesterol [83], which prevents PS from remaining in the intestinal lumen for a long period to inhibit cholesterol absorption. This evidence suggests that phytosterols affect cholesterol absorption, at least in part, by competing with cholesterol for micelles.

Trans-intestinal cholesterol efflux (TICE) refers to the excretion of endogenous cholesterol from the intestinal epithelium into the intestinal lumen via *ABCG5/8* [84]. Approximately 30% of fecal neutral sterols (FNS) in humans are attributed to the action of TICE [85]. In a population-based study, Jakulj et al. found that enhanced TICE action increased fecal cholesterol excretion and accelerated cholesterol turnover [85], thereby reducing circulating cholesterol levels. Notably, PS can also increase the excretion of FNS by enhancing the action of TICE. Nakano et al. found by in vivo experiments that perfusion of intestinal segments with perfusate containing 0.5 mg/mL of phytosterols doubled cholesterol efflux from the brush border membrane (BBM) [86], however, this was not observed when equal amounts of cholesterol were perfused. Lifsey et al. administrated C57BL6/J mice with stigmasterol for 4 days and found that stigmasterol promoted the excretion of neutral sterols and increased the intestinal cholesterol secretion rate [87]. Both cholesterol and PS bind to the BBM upon entering the micelles, but BBM has a limited capacity to accommodate sterols, resulting in excretion of sterols from BBM back into the intestinal lumen. Most of the PS was reabsorbed, and only trace amounts of cholesterol underwent a similar effect. This effect occurs repeatedly through *ABCG5/8* and non-transport protein-mediated efflux on the BBM, leading to increased cholesterol efflux [88]. Thus, PS competes with cholesterol absorption not only in the intestinal lumen, but also on the BBM. In addition to affecting the absorption of cholesterol, phytosterols also have other ways to affect the concentration of circulating cholesterol.

It has been suggested that the mechanism driving this is that phytosterols may reduce cholesterol uptake by intestinal cells by reducing the expression of NPC1L1 in the apical membrane of intestinal cells. However, it was found that plant stanol esters could reduce the absorption of cholesterol in the intestinal tract of hamsters, but that the expression levels of NPC1L1 and ABC sterol transporters were not significantly changed [89]. It was further proven that the mechanism of lowering cholesterol absorption by phytosterols was independent of NPC1L1 and ABC sterol transporters. Juritsch et al. found that, compared with pregnant *ApoE*^−/−^ mice given cholesterol alone [90], the content of total cholesterol in the serum of the pups was significantly reduced in pregnant *ApoE*^−/−^ mice given phytosterols and cholesterol. More importantly, the expression of NPC1L1 and HMGCR was significantly upregulated.

It was then suggested that phytosterols might reduce the amount of cholesterol entering chylomicrons by affecting ACAT2 and MTP. One way for this is that, because phytosterols are poor substrates for ACAT2, phytosterols may compete with cholesterol to reduce esterification [91]. The other way is that phytosterols can reduce the esterification of cholesterol by regulating the expression of ACAT2 and MTP, so as to effectively reduce the amount of cholesterol into chylomicron. Liang et al. fed hamsters with 0.1% β-sitosterol and 0.1% stigmasterol [92], and found that the content of serum total cholesterol decreased significantly, and the mRNA levels of ACAT2 and MTP also decreased. Zhou et al. fed rats with berberine and evodiamine, and four weeks later found that gas chromatography results showed a significant increase in the plasma β-sitosterol content, as well as immunohistochemistry results identifying the expression of ACAT2. What is more, a downward adjustment occurred after the intervention [93]. The above results indicate that phytosterols can reduce the absorption of cholesterol by affecting ACAT2 and MTP, but the exact mechanism is still unclear.

### 5.2. Phytosterols Regulate Liver Cholesterol Metabolism

In order to maintain liver cholesterol homeostasis, dietary intake of phytosterols leads to a decrease in intestinal cholesterol absorption, which will later cause a compensatory increase in the synthesis of endogenous cholesterol in the body. However, Field et al. used β-sitosterol to intervene CaCo-2 cells and found that the expression of the HMGCR gene was suppressed after the intervention [94]. Similarly, Batta et al. fed WKY and Wistar rats with 0.5% stigmasterol and found that the HMGCR activity was reduced by about 75% in WKY rats and about 45% in Wistar rats [95]. Moreover, in mice with hypercholesterolemia, treatment with a plant sterol ester-rich diet did not alter the expression of HMGCR reductase mRNA in the liver and monocytes [96]. This may be due to the post-transcriptional change in HMGCR reductase activity, or the existence of an alternative pathway related to increased cholesterol synthesis.

**Figure 4:**
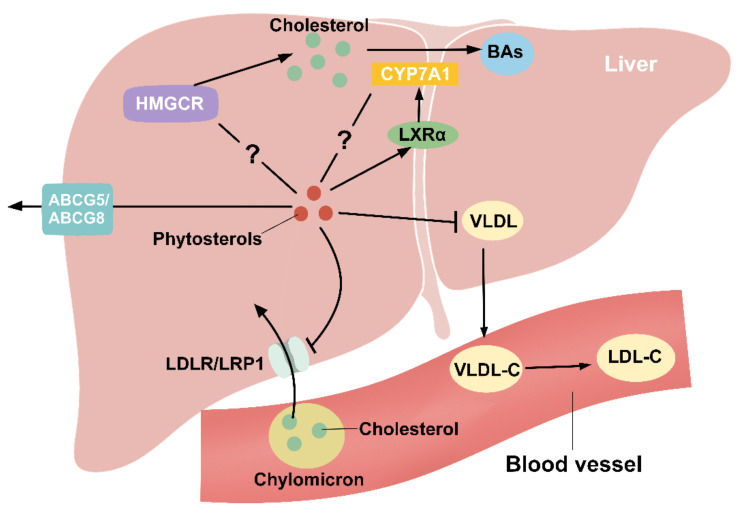
Phytosterols regulate hepatic cholesterol metabolism. Cholesterol in the remnants of chylomicron enters the liver through the action of LDLR and LRP1, which is effectively inhibited by PS. The specific mechanism of phytosterol regulation of HMGCR, a key rate-limiting enzyme for cholesterol synthesis, is unclear. Cholesterol is converted to BAs in the liver by CYP7A1, and LXRα is an upstream regulatory gene of CYP7A1. PS can promote LXRα expression, but the regulatory role for CYP7A1 is controversial. VLDL produced by the liver can be converted to LDL-C in the blood vessels, and PS can decrease hepatic VLDL production to reduce circulating LDL-C levels. PS can also be secreted into the bile via *ABCG5/8*.

The small amounts of phytosterol that enter the circulation are rapidly taken up by the liver and secreted into the bile via hepatic *ABCG5/G8*, further explaining the very low plasma concentration of phytosterols. PS inhibits LDLR and LDL-receptor related protein1 (LRP1) mediated binding and internalization, thereby reducing the entry of cholesterol from the remnants of chylomicron into the liver [81]. Furthermore, phytosterols are found in all lipoproteins with relatively high concentrations in LDL-C and HDL-C [44]. LDL-C is mainly formed in the blood vessels via the conversion of very low density lipoprotein cholesterol (VLDL-C), and phytosterols can reduce hepatic VLDL production [97], indicating that the cholesterol-lowering function of phytosterols may be achieved by reducing the synthesis of VLDLs (Figure 4). It has also been shown that taking 1.5 g of phytosterol equivalents per day can safely and effectively reduce LDL-C levels in healthy adults [41]. Similarly, in patients with metabolic syndrome consuming 4 g of phytosterols daily for two months, the concentrations of TC, TG, LDL-C and small and dense LDL (sdLDL) were significantly reduced [69]. In addition, Demonty et al. showed a dose-response curve in response to phytosterols reducing LDL-C [98], reaching a platform period at 3 g/d, which could thus reduce LDL-C by about 10.7%. On the contrary, intervention using soft capsules containing phytosterols for daily intake of 2 g of phytosterols did not significantly reduce the concentration of TC or LDL-C [99]. It may be speculated that the lack of selection of a proper dose delivery system caused a decrease in the cholesterol-lowering efficacy. In summary, dietary supplementation of phytosterols can not only reduce the absorption of cholesterol in the intestine, but also reduce LDL-C by regulating the production of VLDL in the liver. At the same time, this effect has also shown a dose-response relationship.

He et al. have shown that plant stanol derivatives promote the expression of LXRα and CYP7A1 in the liver and increase the total BAs content in the feces [100]. This suggests that the LXRα-CYP7A1-bile acids excretion pathway may be a potential mechanism for phytosterols to lower cholesterol. In addition, both Lifsey et al. and Cedó et al. found that LXR has a role in regulating cholesterol absorption, but the regulation of cholesterol absorption by phytosterols is independent of intestinal LXR expression [87,101]. Surprisingly, Méndez-González et al. used a modified phytosterol to intervene intestinal and liver cholesterol homeostasis in mice and found that it could upregulate hepatic LXRα but decrease CYP7A1 expression [102]. Therefore, the specific mechanism of the effect of phytosterols on LXRα and CYP7A1 still requires a more in-depth study.

## 6. Conclusions

With the advancement of research, understanding of PS has become more comprehensive and in-depth, which can reduce several risk factors for NAFLD and CVDs, such as TG, TC, and LDL-C. This review summarizes the molecular mechanisms underlying the effects of PS on cholesterol absorption and metabolism in terms of its bioavailability, including competition with cholesterol for absorption in the intestinal lumen and BBM, and regulation of cholesterol metabolism in the liver. However, the cholesterol-regulating effect remains to be further investigated. In addition, there is controversy as to whether PS has a pro-atherogenic effect in vivo, and extensive experimental confirmation is needed. Currently, PS is being used as a dietary supplement for the prevention of chronic diseases associated with hypercholesterolemia, future research should focus on the molecular mechanisms by which PS regulates physiological activities related to cholesterol synthesis, absorption, transport, and metabolism. More, larger population-based multicenter studies, combined with animal studies are required to further demonstrate the efficacy and safety of PS, and shed light on its potential therapeutic and preventive roles and applications in clinical treatment and daily life.

## Figures and Tables

**Table 1:** Cholesterol-reducing potentials of phytosterols in clinical trials.

Study Population	Length of Intervention (Weeks)	Adjustments Considered	The Main Results of Phytosterol Intervention	References
Healthy individuals with slightly higher TG levels (≥1.4 mmol/L) and LDL-C concentrations (≥3.4 mmol/L) (*n* = 260)	4	TG, LDL-C, TC,	Participants in the intervention group had significantly lower concentrations of TC (3.9%), TG (10.6%), and LDL-C (5.2%)	[68]
Patients with metabolic syndrome (*n* = 108)	8	TC, LDL-C, sdLDL, TG	Patients in the intervention group had significantly lower concentrations of TC (15.9%), TG (19.1%), LDL-C (20.3%), and sdLDL (*p* < 0.05)	[69]
Normocholesterolemic participants (*n* = 159)	3	LDL-C	The concentration of LDL-C (5.96%, *p* = 0.028) was significantly lower in patients in the intervention group	[42]
The fasting TC concentration of the participants was 6.57 ± 0.13 mmol/L (*n* = 70)	4	TC, LDL-C	Patients in the intervention group had significantly lower concentrations of TC (4.8%, *p* < 0.05) and LDL-C (8.1%, *p* < 0.05)	[70]
Healthy individuals at increased risk of T2DM and patients with T2DM (*n* = 161)	6	TC, LDL-C, TG	Individuals in the phytosterol intervention group had significantly lower fasting TC (4.2%), TG (8.3%), and LDL-C (4.6%) concentrations	[71]
Postmenopausal women (*n* = 38)	6	TC, LDL-C	Serum TC (212.9 ± 25.8 mg/dL) and LDL-C concentrations (121.7 ± 24.4 mg/dL) decreased significantly after phytosterol treatment compared to previous (220.0 ± 27.8 mg/dL) (129.4 ± 28.5 mg/dL)	[72]
Individuals not taking cholesterol-lowering drugs or without diabetes (*n* = 221)	3	TC, LDL-C, diastolic blood pressure	Serum LDL-C concentration (9.5 ± 2%), TC (*p* < 0.01), and diastolic blood pressure (*p* = 0.01) were significantly reduced after phytosterol intervention	[73]

TG, triglycerides; TC total cholesterol; LDL-C, low density lipoprotein cholesterol; sdLDL, small and dense low density lipoprotein.

## Data Availability

Not applicable.
